# Bullous Aplasia Cutis Congenita—Description of a Novel Dermoscopic Feature

**DOI:** 10.5826/dpc.1102a12

**Published:** 2021-03-08

**Authors:** Aradhana Rout, Sandeep Arora, Rajeshwari Dabas, Debatraya Paul

**Affiliations:** 1 Department of Dermatology, Command Hospital Air Force Bangalore, India

**Keywords:** bullous aplasia cutis congenita, cobblestone pattern, developmental abnormality, congenital anomalies, dermoscopy

## Case Presentation

**Figure 1:**
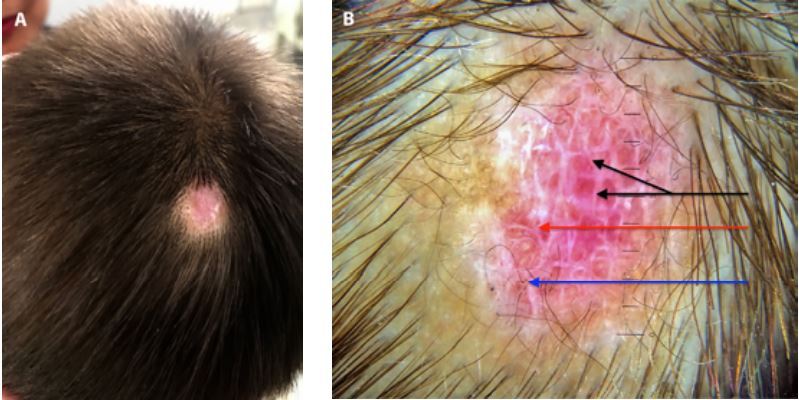
(A) Solitary alopecic patch with atrophic translucent skin and the (B) dermoscopic image with absent follicular openings, a few distended blood vessels (red arrow), a few visible pigmented hair bulbs (blue arrow), and cobblestoning (black arrows).

A 1-year-old boy presented with a solitary alopecic atrophic area on the scalp and a history of a fluid-filled lesion at the site that had presented a few days after birth and healed with thinning of skin (Figure 1A). Examination revealed a 2 × 2 cm solitary, well-defined alopecic patch with atrophy on the scalp vertex without any underlying bone defect.

Dermoscopy revealed (Figure 1B) absent follicular openings, a few distended blood vessels, a few visible hair bulbs, and a branching network of reticulated white-colored streaks on atrophic skin resembling cobblestones.

## Teaching Point

Although all signs may not be present in a case, the classic dermoscopic findings described are [1]: absence of follicular openings, thick, distended blood vessels, a hair collar sign with hair shafts arranged radially and forming a ring of hypertrichosis, and bulbs of anagen hair seen through the translucent epidermis resembling a golf stick [2]. However, recently a new pseudomembranous pattern has been identified, which can be included within a clinical and dermoscopic spectrum ranging from the classic to the pure membranous form of aplasia cutis congenita [3]. Of these findings, our case had only a few distended vessels but was absent hair collar and golf stick signs. However, the atrophic skin in our case presented as cobblestoning visible on dermoscopy, which is a unique feature of bullous aplasia cutis congenita.
